# Hyponatremia Promotes Cancer Growth in a Murine Xenograft Model of Neuroblastoma

**DOI:** 10.3390/ijms242316680

**Published:** 2023-11-23

**Authors:** Giada Marroncini, Laura Naldi, Benedetta Fibbi, Alice Errico, Simone Polvani, Marco Brogi, Alessandra Fanelli, Mario Maggi, Alessandro Peri

**Affiliations:** 1 Endocrinology, Department of Experimental and Clinical Biomedical Sciences “Mario Serio”, University of Florence, AOU Careggi, 50139 Florence, Italy; giada.marroncini@unifi.it (G.M.); laura.naldi@unifi.it (L.N.); alice.errico@stud.unifi.it (A.E.); mario.maggi@unifi.it (M.M.); alessandro.peri@unifi.it (A.P.); 2 Pituitary Diseases and Sodium Alterations Unit, Careggi University Hospital, 50139 Florence, Italy; 3 Gastroenterology Unit, Department of Experimental and Clinical Biomedical Sciences “Mario Serio”, University of Florence, 50139 Florence, Italy; 4 Central Laboratory, Careggi University Hospital, 50139 Florence, Italy; brogima@aou-careggi.toscana.it (M.B.); fanellia@aou-careggi.toscana.it (A.F.)

**Keywords:** hyponatremia, Syndrome of Inappropriate Antidiuresis, cancer, neuroblastoma, murine xenograft

## Abstract

In cancer patients, hyponatremia is detected in about 40% of cases at hospital admission and has been associated to a worse outcome. We have previously observed that cancer cells from different tissues show a significantly increased proliferation rate and invasion potential, when cultured in low extracellular [Na^+^]. We have recently developed an animal model of hyponatremia using Foxn1nu/nu mice. The aim of the present study was to compare tumor growth and invasivity of the neuroblastoma cell line SK-N-AS in hyponatremic vs. normonatremic mice. Animals were subcutaneously implanted with luciferase-expressing SK-N-AS cells. When masses reached about 100 mm^3^, hyponatremia was induced in a subgroup of animals via desmopressin infusion. Tumor masses were significantly greater in hyponatremic mice, starting from day 14 and until the day of sacrifice (day 28). Immunohistochemical analysis showed a more intense vascularization and higher levels of expression of the proliferating cell nuclear antigen, chromogranin A and heme oxigenase-1 gene in hyponatremic mice. Finally, metalloproteases were also more abundantly expressed in hyponatremic animals compared to control ones. To our knowledge, this is the first demonstration in an experimental animal model that hyponatremia is associated to increased cancer growth by activating molecular mechanisms that promote proliferation, angiogenesis and invasivity.

## 1. Introduction

Hyponatremia is the most common electrolyte disorder encountered in hospitalized patients. In this setting, the prevalence of hyponatremia is about 30%. The same considerations apply to cancer patients, in which low serum sodium ([Na^+^]) is present in around 40% of cases at admission [1]. In addition, roughly half of cancer patients experience one or more episodes of hyponatremia during their disease. In principle, each type of cancer can be associated to hyponatremia, although this alteration is most often present in lung cancer [2]. The main cause of hyponatremia in oncology is the Syndrome of Inappropriate Antidiuresis (SIAD), which is often related to ectopic secretion of arginine vasopressin (AVP) by tumoral cells [3,4]. However, in cancer patients SIAD may be secondary also to the administration of drugs that stimulate AVP secretion (e.g., anticancer drugs, opioids, non-steroidal anti-inflammatory drugs, antidepressants). Non SIAD-related hyponatremia may also occur, as a consequence for instance of vomiting, diarrhea, hydration during chemotherapy, diuretics administration, presence of comorbidities [3,4].

Interestingly, hyponatremia has a negative impact on both Progression-Free Survival (PFS) and Overall Survival (OS) in many types of tumors, including lung, gastrointestinal, liver, renal, prostatic, pancreatic, genitourinary cancer, mesothelioma and lymphoma [1]. In a large series of patients admitted to a University Cancer Center, an almost three-fold higher hazard ratio for death in hyponatremic patients than in normonatremic ones was observed [5]. Conversely, there is evidence that hyponatremia correction ameliorates patients’ outcome [6,7,8].

We have previously established an in vitro model of hyponatremia [9]. An extensive microarray analysis indicated that in human neuroblastoma cells the expression of more than 40 genes was markedly affected upon cells exposure to low [Na^+^]. These genes could be clustered in different groups, which were related to cell proliferation and motility [9]. Noteworthy, the gene that showed the highest increase in the expression level in low [Na^+^] was the heme oxigenase-1 (HMOX-1) gene. HMOX-1 has an antioxidant activity that represents a response to oxidative stress and is also associated with anti-apoptotic effects [1].

More recently, we have confirmed the presence of HMOX-1 overexpression in different cancer cell lines from lung, pancreas, colorectal cancer, neuroblastoma and chronic myeloid leukemia, when grown in low [Na^+^] [10,11]. We also found that in this condition cell proliferation and invasivity significantly increased, whereas apoptotic death was blunted. Accordingly, an overactivation of the RhoA, ROCK-1, ROCK-2 pathway, which is involved in cell growth and invasion, was observed. In addition, we detected an altered expression of cytoskeleton-associated proteins that lead to actin cytoskeletal remodelling and cell motility [10,11].

To our knowledge, no data regarding cancer growth in animal models of hyponatremia have been published, so far. We have recently developed an animal model of hyponatremia, by administering desmopressin (dDAVP) via osmotic minipumps to Foxn1^nu/nu^ mice [12].

The aim of the present study was to take advantage of this murine model of hyponatremia, in order to compare tumor growth and invasivity of neuroblastoma cells (i.e., SK-N-AS cell line, one of the cell lines we have previously tested in in vitro studies) in hyponatremic vs. normonatremic animals.

## 2. Results

### 2.1. Induction of Hyponatremia in Nude Mice

**Figure 1:**
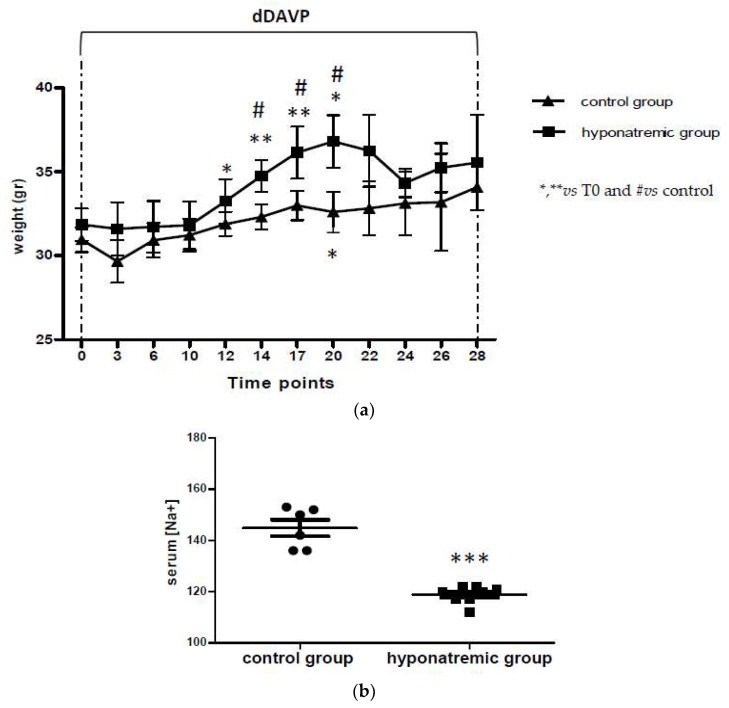
Body weight and serum [Na^+^]. (**a**) Body weight fluctuations in the two experimental groups. Results are expressed as mean ± SE. * *p* ≤ 0.05, ** *p* ≤ 0.02 vs. T0 and ^#^ *p* ≤ 0.05 vs. control group. (**b**) Serum [Na^+^] (mEq/L) in control group and hyponatremic group. Results are expressed as mean ± SE. *** *p* ≤ 0.002 vs. control group.

Foxn1^nu/nu^ mice were subcutaneously implanted with 2 × 10^6^ SK-N-AS Luc2-positive cells on both flanks. When masses of about 100 mm^3^ were reached (T0), hyponatremia was induced as previously described [12] for 28 days with dDAVP infusion at the rate of 0.3 ng/h. According to previous findings, the hyponatremic group (*n* = 8) showed a significant increase of weight from T12 to T20, with a significant difference vs. the normonatremic (i.e., control) group (*n* = 6) (Figure 1a). However, after T20, the body weight of animals in the hyponatremic group and in the control group were similar, likely by compensatory mechanisms. For the control group, no significant weight difference was observed during the experimental period, with the exception of an increase at T17. At sacrifice, in accordance with previous results, serum [Na^+^] was significantly lower in dDAVP treated mice (118.69 ± 2.15 mEq/L, mean ± SE), compared to controls (145.42 ± 3.36 mEq/L, mean ± SE, *p* ≤ 0.002), (Figure 1b, Table 1).

### 2.2. Analysis of Tumor Masses and Survival

**Figure 2:**
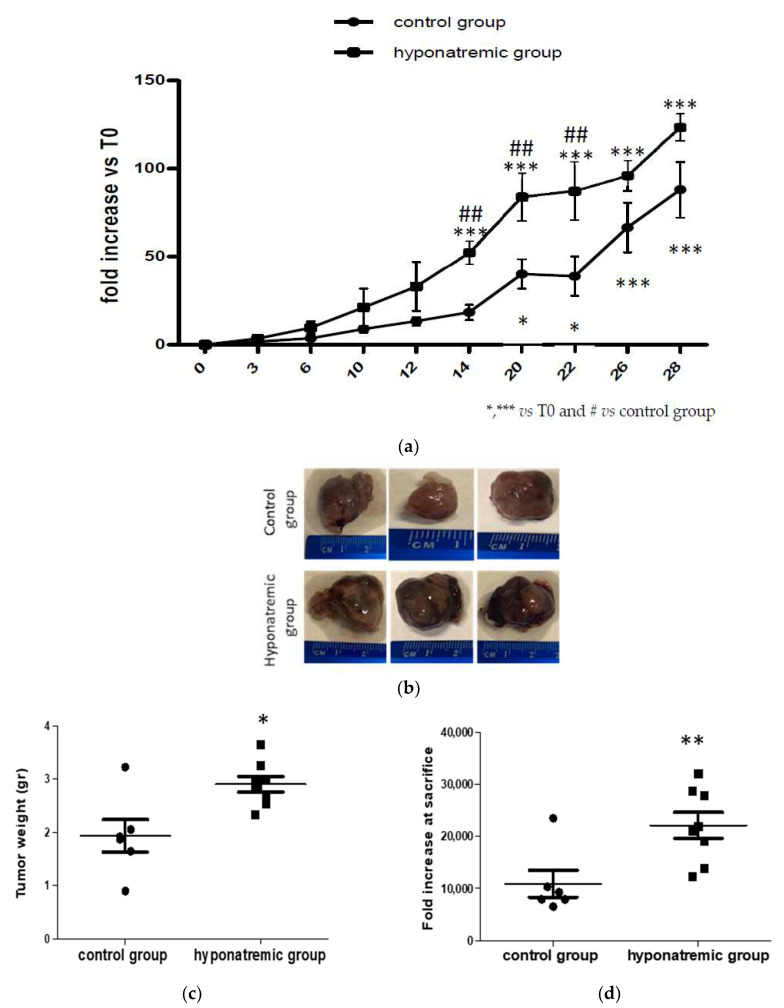
Analysis of tumor growth. (**a**) Tumor masses in control and hyponatremic groups were measured at different time points. Results are expressed as mean ± SE. * *p* ≤ 0.05, *** *p* ≤ 0.002 vs. T0 and ^##^ *p* ≤ 0.05 vs. control group. (**b**) Representative examples, (**c**) weight (gr) and (**d**) volume (mm^3^) of explanted tumor masses. Results are expressed as mean ± SE. * *p* ≤ 0.05, ** *p* ≤ 0.02 vs. control group.

Tumor masses were measured independently by two different operators at each time point and the median values (reported in Table S1 of Supplemetary Materials) were considered. In both the control and the hyponatremic group tumor mass volume was expressed as fold increase compared to T0. Differences in tumor growth were early observed. Notably, starting from T6 the slope of growth curves in the two experimental groups differed and the difference became statistically significant from T14 to T22 (*p* ≤ 0.05) (Figure 2a). At sacrifice, tumor masses were explanted and weight and volume were measured (Figure 2b–d). Tumor mass weight and volume at the day of sacrifice were significantly greater in the hyponatremic group (*p* ≤ 0.05 and *p* ≤ 0.02 vs. control, respectively).

**Figure 3:**
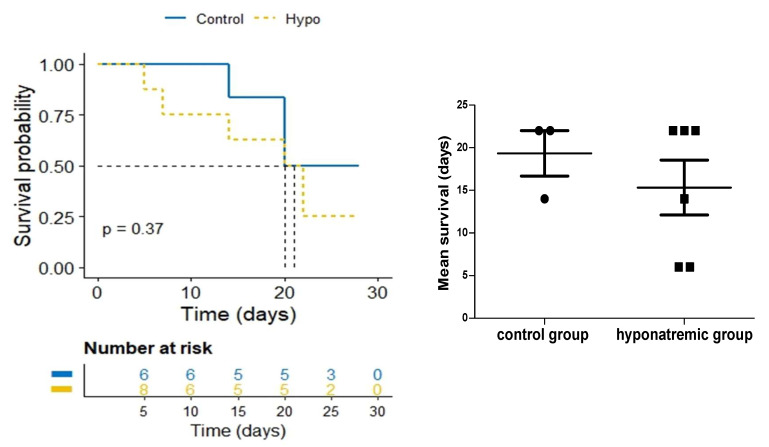
Average survival. Kaplan-Meier survival analysis of control and hyponatremic mice. The bar graph shows the mean survival values and the dotted line represents the median. Results are expressed as mean ± SE.

Kaplan-Meier survival analysis showed a trend to a lower survival of hyponatraemic mice, with a median survival time of 15.3 ± 3.22 days (mean ± SE) compared to and 19.3 ± 2.67 days (mean ± SE) in the control group (Figure 3).

### 2.3. Analysis of Tumor Progression with IVIS Lumina 5 System

**Figure 4:**
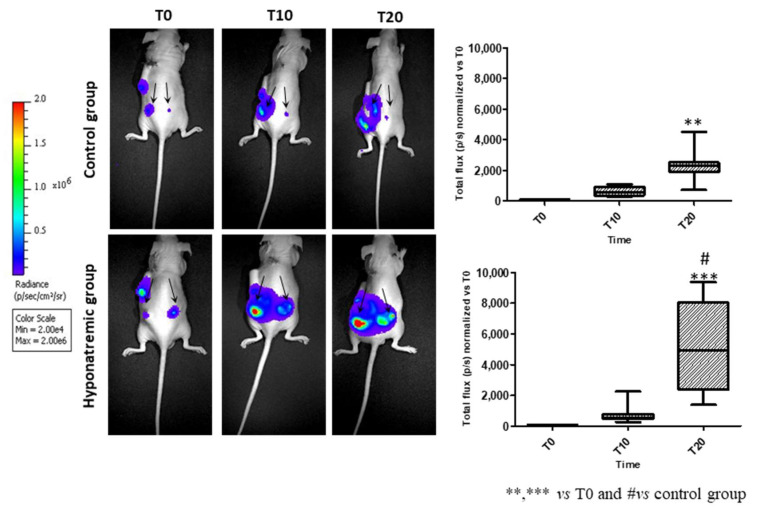
In vivo imaging. Representative images of bioluminescence imaging after intraperitoneal injection of 100 µL/10 gr Luciferin of one control and one hyponatremic mouse at different time points. Bar graphs represent total flux (p/s) of bioluminescence emissions of tumor masses (indicated by arrows) in control group vs. hyponatremic group. Results are expressed as mean ± SE. ** *p* ≤ 0.02 and *** *p* ≤ 0.002 vs. T0 and **^#^** *p* ≤ 0.05 vs. control group.

Tumor activity was assessed in vivo with the IVIS Lumina S5 System at different time points: T0, T10 and T20. At T20, the bioluminescence emission was significantly increased both in the control and in the hyponatremic group, when compared to T0 (*p* ≤ 0.02 and *p* ≤ 0.002 vs. T0). More interestingly, at T20 the bioluminescent emission of tumor masses in the hyponatremic group was significantly greater than in the control one (*p* ≤ 0.05) (Figure 4).

### 2.4. Histological and Immunohistochemical Analysis

**Figure 5:**
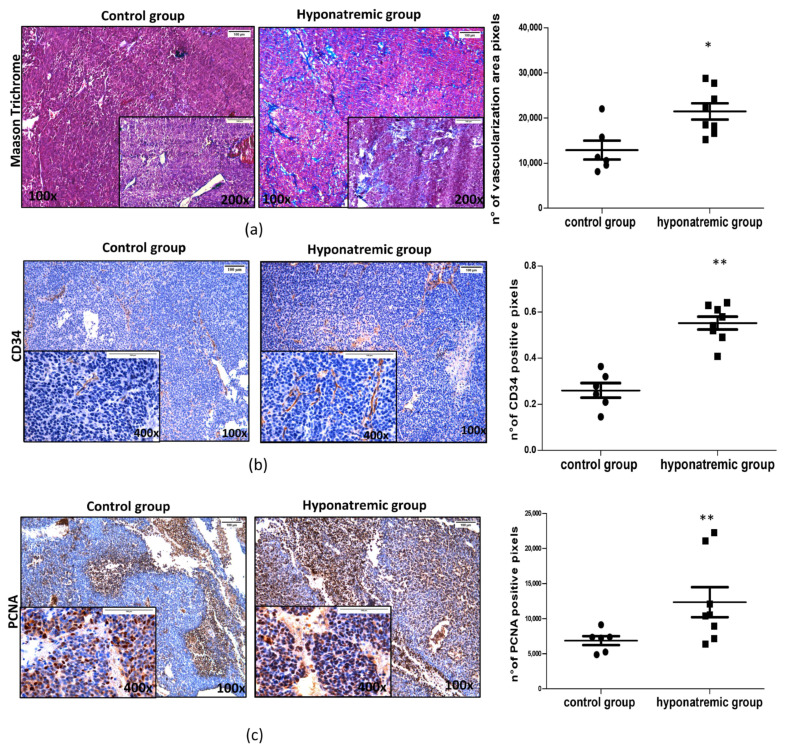

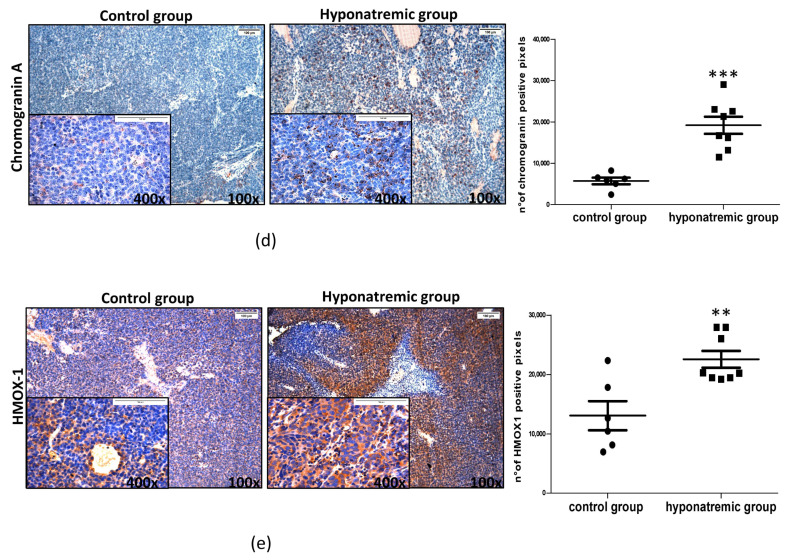
Masson’s Trichrome and immunohistochemical staining of tumor masses. Representative images of tumor masses sections of one control and one hyponatremic mouse. (**a**,**b**). Increased vascularization is evidenced by Masson’s Trichrome staining and immunohistochemical analysis for CD34. In the bar graphs, densitometric analysis of positive pixels of vascularization area (aniline blue positive cells) and CD34 positive cells are represented. Results are expressed as mean ± SE. * *p* ≤ 0.05, ** *p* ≤ 0.02 vs. control group. (**c**) Immunohistochemical analysis of PCNA. In the bar graph, densitometric analysis of positive pixels of PCNA positive cells is represented (** *p* ≤ 0.02 vs. control group). (**d**) Immunohistochemical analysis of chromogranin A. In the bar graph, densitometric analysis of positive pixels of chromogranin A positive cells is represented (*** *p* ≤ 0.002 vs. control group). (**e**) Immunohistochemical analysis of HMOX-1. In the bar graphs, densitometric analysis of positive pixels of HMOX-1 positive cells is represented (** *p* ≤ 0.02 vs. control group). All results are expressed as mean ± SE.

Paraffine embedded tumor masses were histologically and immunohistochemically analyzed. Masson’s Trichrome staining showed that in hyponatremic mice tumors were more vascularized than in normonatremic animals (*p* ≤ 0.05 vs. control group) (Figure 5a). This finding was confirmed by immunohistochemical analysis for CD34, a marker of endothelial progenitor cells, which was found to be more intensively expressed in the tumor masses of hyponatremic mice (*p* ≤ 0.02 vs. control group) (Figure 5b). The levels of expression of the proliferating cell nuclear antigen (PCNA) were also significantly greater in the tumor masses of hyponatremic mice compared to normonatremic mice (*p* ≤ 0.02 vs. control group) (Figure 5c). Chromogranin A, a protein widely expressed in neuroblastomas that has a strong correlation with cell proliferation, showed a significantly stronger expression in tumors from hyponatremic mice (*p* ≤ 0.002 vs. control group) (Figure 5d). The expression of the oxidative marker protein HMOX-1 was also analyzed. Again, HMOX-1 was more intensively expressed in the masses of hyponatremic mice (*p* ≤ 0.02 vs. control group) (Figure 5e).

Interestingly, we found a significant correlation between tumor volume in hyponatremic animals and molecular markers that were analysed (Figure S1a). A relationship, yet not statistically significant, was also observed between animal survival and CD34 and PCNA expression (Figure S1b), as shown in Supplementary Materials.

### 2.5. Analysis of MMPs Activity In Vivo with IVIS Lumina S5

**Figure 6:**
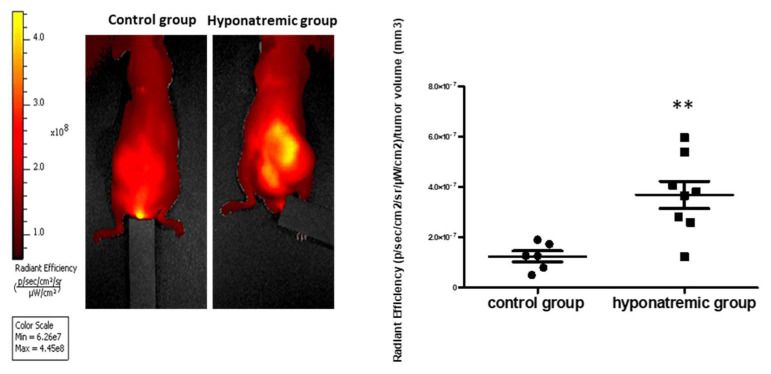
Metalloprotease activity. Fluorescent imaging after vein injection of 100 µL MMPSense^TM^ probe at the day of sacrifice. Representative images of one control and one hyponatremic mouse are shown. Bar graph represents total flux (p/s)/tumor volume (mm^3^) of fluorescent emissions of tumor masses in control group vs. hyponatremic group. Results are expressed as mean ± SE. ** *p* ≤ 0.02 vs. control group.

The day of sacrifice, the activity of MMPs, proteins related to tumor aggressiveness and metastatic potential, was analyzed. MMPs were significantly more expressed in the tumor masses of hyponatremic mice compared to the control group (*p* ≤ 0.05 vs. control group) (Figure 6).

## 3. Discussion

The awareness that hyponatremia may represent a negative prognostic factor in different pathologies has increased in the last decennium [13]. Experimental and clinical evidence clearly indicate that this apply also to cancer [1]. Interestingly, pre-treatment low serum [Na^+^] have been related to a worse response to chemotherapy [14,15]. Noteworthy, besides its negative prognostic role, hyponatremia has been associated to an increased length of stay in the hospital and to a greater economic burden [16].

In order to further clarify the role of hyponatremia on tumoral growth, in this study we used a mouse model of hyponatremia secondary to SIAD, previously developed in our laboratory [12], and we created a xenograft model of neuroblastoma.

In agreement with previous in vitro findings [10,11], we found that the growth of tumoral lesions was significantly greater in hyponatremic animals. The different growth vs. normonatremic mice was observed starting from a few days after the induction of hyponatremia and was maintained until the end of the experimental protocol. In addition to tumor size and weight, differences among the groups were also clearly visualized using the IVIS Lumina 5 System, which is based on bioluminescence emission.

Furthermore, in agreement with the many clinical observations that both PFS and OS are reduced in hyponatremic cancer patients [1,2], we observed a trend to a reduced survival in hyponatremic mice compared to normonatremic ones.

In order to investigate on the cellular alterations leading to an increased growth of the tumoral lesions in hyponatremic animals, we performed additional analyses on histological sections. Interestingly, we found that the vascularization of tumor lesions obtained from hyponatremic mice was more evident than in normonatremic animals. It is very well known that the growth of solid tumors is closely related to the recruitment of blood vessels. The idea of tumor angiogenesis was first proposed by Judah Folkman in 1971 [17]. Tumor cell survival is warranted by the expression of pro-angiogenic factors, which induce the generation of new vessels [18]. In turn, blocking angiogenesis has been identified as a possible pharmacological strategy in cancer treatment [19]. The CD34 protein was first identified about four decades ago as a biomarker of hematopoietic stem cells [20], but its expression has been more recently observed also in other cell types, including endothelial precursors, which are actively involved in blood vessels formation [21].

Thus, CD34 has been also utilized as a biomarker to assess angiogenesis in malignancies [22] and an increased number of CD34 surface-expressing cells has been correlated with disease progression and therapy resistance in neuroblastoma [23]. Noteworthy, the immunostaining for CD34 was more intense in the vessels of tumor lesions from hyponatremic mice, with a statistically significant difference. In addition, we found a significantly higher expression of the PCNA in this group of animals. PCNA is a nuclear protein, which is involved in DNA replication, elongation and repair [24]. PCNA also regulates cell cycle progression through the G1/S boundary by interacting with cyclin/cdk, and it has been identified as a possible target for anticancer strategies [24,25,26,27,28].

Immunohistochemical analysis also revealed the presence of a more intense staining for chromogranin A in tumor samples from the hyponatremic group. Chromogranin A is a 456-amino acid protein of the granin family, which is expressed in endocrine, neuroendocrine, peripheral and central neural tissues [29]. Serum levels of chromogranin A are elevated in different neuroendocrine tumors, including carcinoids, pancreatic tumors, pheochromocytoma, paraganglioma, and neuroblastoma [30]. Interestingly, serum chromogranin A levels in patients with neuroblastoma are associated with a worse outcome [31,32] and patients with advanced disease stages have higher serum levels than those with localized disease [31]. It has been reported that the reduction of chromogranin A levels by knockout approaches in neuroblastoma cells caused a reduced cell proliferation rate by inhibiting the AKT/ERK pathway, whereas in an in vivo xenograft model of neuroblastoma chromogranin A knockdown led to a more differentiated (S-type) phenotype, which is known to be associated to a more favourable outcome [33].

In the lesions excised from hyponatremic mice we also detected higher levels of expression of the HMOX-1 gene, compared to control animals. It is worth mentioning that HMOX-1 has been associated to several functions that overall create a microenvironment that favors tumor growth. In particular, it has been shown to promote carcinogenesis, cell proliferation, angiogenesis and invasion. It has been also demonstrated that HMOX-1 can induce chemoresistance by limiting Reactive Oxygen Species-mediated oxidative damage, promoting apoptosis resistance and activating protective autophagy [34,35]. For these reasons, HMOX-1 has been considered as a possible target in anticancer strategies and in vitro as well as in vivo observations reported that selective inhibition of HMOX-1 reduces cell proliferation and invasion, whereas it induces cell apoptosis [34,35,36]. Admittedly, overall these histological data are further validated by the direct relationship we observed between them and tumor volume in hyponatremic animals.

Finally, we detected an increased amount of MMPs in tumor masses from hyponatremic mice. This finding is in agreement with similar data observed in cancer cells grown in low extracellular [Na^+^] [10,11]. Matrix metalloproteases represent a category of proteolytic enzymes that have a fundamental role in extracellular matrix degradation. As such, they participate in multiple physiological and pathological processes, including cancer. Here, MMPs favor tumor progression not only by degrading matrix proteins, but also by modulating the immune response and influencing the tumor microenvironment [37,38].

Overall, the present results represent the first demonstration in an experimental animal model that hyponatremia is associated to cancer growth by activating molecular mechanisms that lead to increased proliferation, angiogenesis and invasivity. As previously reported in in vitro experiments [10,11], hyponatremia-related modulation of oxidative stress appears to have an important role in favoring cancer progression. These data are in agreement with the robust clinical observations that hyponatremia is associated to a worse outcome in cancer patients and further reinforce the recommendation to promptly recognize and correct this electrolyte alteration. This recommendation is strengthened by clinical reports indicating that the normalization of serum [Na^+^] in cancer patients results in a prolonged PFS and OS [6,7,8].

Whether hyponatremia might be viewed also as a risk factor to develop cancer remains an unsolved question, so far. However, it is worth mentioning that two large retrospective cohort Danish studies, which identified patients with a first-time diagnosis of hyponatremia in medical registries, showed that low serum [Na^+^] was associated to a significantly increased risk to have a subsequent diagnosis of cancer [39]. The authors suggest that hyponatremia might be a marker of occult neoplasms [40]. These findings, which need to be confirmed by additional studies, suggest that a correct [Na^+^] balance might be seen in the future also as a prevention measure against cancer, in addition to a marker of prognosis.

## 4. Materials and Methods

### 4.1. Chemicals and Reagents

Human stromal type neuroblastoma tumor cells (SK-N-AS, Manassas, VA, USA), Dulbecco’s Modified Eagle Medium (DMEM) culture medium, fetal bovine serum (FBS), L-glutamine and antibiotics (penicillin and streptomycin), Hank’s Balanced Salt Solution (BSS) were purchased from Millipore (Milan, Italy).

### 4.2. Cell Cultures and Cell Transfection

SK-N-AS cells were cultured in DMEM supplemented with 10% FBS, L-glutamine and antibiotics (50 U/mL penicillin and 50 μg/mL streptomycin) and maintained at 37 °C in a humidified atmosphere (5% CO_2_/95% air).

Luciferase-expressing SK-N-AS cells were produced as follows. Two million cells were cultured in six-well plates with 1.6 mL of DMEM; upon reaching 80% of confluence, cells were transfected with pGL4.51(Luc2/CMV/Neo) plasmid (Promega Corporation, Madison, WI, USA), an engineered vector containing Luc2 sequence for luciferase expression in mammalian cells and the gene for geneticin resistance. Transfection occurred using the commercial Effectene^®^ Transfection Reagent kit (301425, QIAGEN, Hilden, Germany) operating as per protocol. Briefly, 4.0 µg of plasmid DNA was suspended in 100 µL of 1× Tris-EDTA buffer with 3.2 µL of Enhacer. After 2–3 min of incubation at room temperature, 10 µL of Effectene^®^ Transfection Reagent was added to the mix, which was dispensed dropwise into each well. After 72 h of growth in the transfection medium, cells were washed with 1× PBS and placed in complete medium with the addition of geneticin (G418, 108321-42-2, Invivogen, San Diego, CA, USA). The optimal concentration of 800 µg/mL used for cell clone selection was identified by treating cells with increasing doses of G418 (0–1000 µg/mL).

### 4.3. A Murine Xenograft Model of Neuroblastoma

All animal experiments were conducted in accordance with institutional ethical standards and national laws after approval by the Ministry of Health [D. No. 512/2022-PR (prot. 17E9C.261)]. Eight-week-old male Foxn1nu/nu mice (*n* = 14) (Charles River Laboratories International, Wilmington, MA, USA) were housed in sterile areas equipped with ventilation and sterile barriers with a 12/12-h light/dark cycle and a constant temperature (21–23 °C) in a standard animal facility (Ce.S.A.L., Department of Biomedical, Experimental and Clinical Sciences “Mario Serio,” Florence, Italy), inside “sterile filter top” cages. In the first week of acclimatization, all mice had ad libitum access to standard chow (MF^®^; Oriental Yeast Co., Ltd., Tokyo, Japan) and tap water. After one week they were subcutaneously implanted with 2 × 10^6^ SK-N-AS Luc2-positive cells on both flanks. Tumor masses were monitored daily using a digital caliper and the volume (mm^3^) was calculated using the following formula: 0.52 × long side × (short side)^2^. Upon reaching a volume masses of about 100 mm^3^, hyponatremia was induced as previously described [29] by subcutaneous implantation of an osmotic minipump (model 1004, Alzet, Cupertino, CA, USA). Mice were randomly divided into two experimental groups: a control group (*n* = 6), implanted with isotonic saline-charged minipumps (0.9% NaCl), and a treatment (i.e., hyponatremic) group (*n* = 8), implanted with dDAVP-filled minipumps (MINIRIN/DDAVP 0.05 mg/mL, Ferring S.P.A., Milan, Italy); in both groups the flow rate was 0.3 ng/h, for 28 days. To keep ingested fluid intake controlled, for the entire duration of the experiment the treatment group was fed with rodent liquid diet only, without access to tap water. The control group was fed with the same liquid diet and had free access to tap water. The animals were sacrificed at day 28 or when the human end point was reached (cachexia, loss of weight ≥ 20%, epilepsy, inability to move, tumor ulceration), according to the Italian Health Ministry protocol.

### 4.4. In Vivo Imaging: IVIS Lumina S5 Imaging System

Tumor growth was assessed from the day of mini-pumps implantation (time point zero, T0), every 10 days (T10, T20) and until the day of sacrifice, using the IVIS Lumina S5 imaging system (Perkin Elmer, Waltham, MA, USA). Before imaging, 100 µL/10 g of body weight of D-luciferin potassium salt solution (15 mg/mL, Perkin Elmer, Waltham, MA, USA) was intraperitoneal injected into each mouse. Three to five minutes post-injection mice were anesthetized with 2.5% isoflurane (1 L/min flow); bioluminescent images were acquired 15 min after initial injection with a Lumina IVIS S5, provided by the Department of Experimental and Clinical Biological Sciences “Mario Serio” (Florence, Italy). Luminescence was measured as Radiance (total Flux photon/sec) with the Living Image^®^ 4.7.2 Software (Perkin Elmer, Waltham, MA, USA) in the region of interest (ROI) encompassing tumor masses.

For fluorescent imaging of metalloproteases (MMPs) activity, 24 h before sacrifice MMPSense™ 750 FAST fluorescent probe (100 µL for each animal, Perkin Elmer, Waltham, MA, USA) was administered into the tail vein. Fluorescent images were acquired with optimal filter for the dye with a Spectral unmixing protocol to reduce autofluorescence signalling. Fluorescence, corrected by the spectral unmixing protocol, was measured as “Radiant Efficiency” (p/s/cm^2^/sr/μW/cm^2^) with the Living Image^®^ 4.7.2 Software (Perkin Elmer, Waltham, MA, USA) in region of interest (ROI) encompassing tumor masses.

### 4.5. Serum [Na^+^] Analysis

Animals were sacrificed with an overdose of anaesthetic (ketamine/xylazine) to allow beating heart blood sampling by transthoracic cardiocentesis to analyze serum [Na^+^]. Blood samples were centrifuged at 3000× *g* for 10 min at +4 °C and processed for [Na^+^] measurement using the Cobas 8000 (Roche/Hitachi family, Basel, Switzerland). Biochemical analyses were carried out by the General Clinical Chemical laboratory of AOU Careggi (Florence, Italy), according to the standard procedures.

### 4.6. Tissues Preparation and Morphological Characterization

At sacrifice, tumor masses were rapidly explanted. Tumor masses were measured and weighted and fixed in 10% formalin (65-30001F—Bio-Optica Milano Spa, Milan, Italy) for at least 48 h and washed twice in water before embedding in paraffin (ASP300S and HistoCore processor, Arcadia Inclusion System, Leica Biosystems, Milan, Italy). Tumor masses sections (5–7 µm) were stained with hematoxylin and eosin (Hematoxylin Gill 3, 05-06015L and Eosin Y alcoholic solution, 05–10003/L-Bio-Optica Milano Spa, Milan, Italy) and finally all slides were dehydrated and mounted in a resinous medium (09-00500, Eukitt-BioOptica Milano Spa, Italy).

### 4.7. Masson’s Trichrome Analysis

Masson’s Trichrome was proceeded as described in the manufacture instruction (14–118—DDK Italia S.r.l., Milan, Italy). After de-paraffinization and rehydration, formalin-fixed slices were placed in bouin’s liquid for 1 h at 56 °C and washed in running water to clean the sections. The slices were stained with Waigert’s hematoxylin for 10′ and washed in tap water. Subsequently, they were stained with biebrich scarlet-acid fuchsin for 15′ and, after another passage in tap water, stained with phosphotunstic acid for 10–15′ and aniline blue for 1–2′. Aniline blue positive pixels were analyzed and quantified using ImageJ (https://fiji.sc (accessed on 5 August 2020) and GraphPad Prism 5.0 Software (https://www.graphpad.com (accessed on 16 April 2021)).

### 4.8. Immunohistochemical Analysis

After de-paraffinization and rehydration, formalin-fixed slices were boiled in Buffer Citrate (pH = 6) at 95 °C for 10 min for antigenic unmasking, placed in 6% H_2_O_2_ solution for 30 min at room temperature to inhibit tissue peroxidases and blocked in PBS/BSA 2% solution for 1 h. To reduce endogenous antibodies binding, slices were incubated with ReadyProbes™ Mouse-on-Mouse IgG Blocking Solution (R37621, Invitrogen, Waltham, MA, USA) for 1 h at room temperature. After that, tissue sections were incubated with the following primary antibody: rabbit polyclonal anti-HMOX1 (ab52947, 1:100, Abcam, Cambridge, UK), mouse monoclonal anti-PCNA (#2586, 1:16,000, Cell Signaling Technology, Danvers, MA, USA), mouse monoclonal anti-chromogranin A (MA5-13096, 1:800, Invitrogen, Waltham, MA, USA), mouse monoclonal anti-CD34 (ab8158, 1:50, Abcam, Cambridge, UK) at 4 °C overnight.

After one-hour incubation with the specific secondary antibody conjugated to horseradish peroxidase (HRP-linked anti-mouse IgG, #7076 or HRP-linked anti-rabbit IgG, #7074 Cell Signaling Technology, Danvers, MA, USA), SignalStain^®^ DAB Substrate Kit (#8059, Cell Signaling Technology, Danvers, MA, USA) were used for antigen detection. DAB positive cells were analyzed and quantified using ImageJ (https://fiji.sc (accessed on 5 August 2020)) and GraphPad Prism 5.0 Software (https://www.graphpad.com (accessed on 16 April 2021).

### 4.9. Statistical Analysis

Each experiment was performed in triplicates, unless otherwise stated. Statistical analysis was performed with GraphPad. Normality of data distribution was assessed with the Shapiro–Wilk normality test. When comparing multiple groups, ANOVA followed by Dunn’s test was used for parametric data, whereas the Kruskal–Wallis test followed by the Conover-Iman test was used for pairwise comparisons of non-parametric data. Values were expressed as mean ± standard error (SE), and *p* ≤ 0.05 was considered to indicate a statistically significant difference. Correlation analysis of histological markers with tumor volume and survival in hyponatremic mice was evaluated by SPSS 28.0.1.0(142) software, considering *p* ≤ 0.05 as statistically significant.

## Figures and Tables

**Table 1:** Serum [Na^+^] in control and hyponatremic mice, as measured at the time of sacrifice.

Time Point of Sacrifice	T6	T14	T22	T28
Number of control mice sacrificed	/	*n* = 1	*n* = 2	*n* = 3
[Na^+^] serum of control mice	/	153	136 150	152 142 136
Number of hyponatremic mice sacrificed	*n* = 2	*n* = 1	*n* = 3	*n* = 2
[Na^+^] serum of hyponatremic mice	122 122	119.88	121 120 117	117 112

## Data Availability

The data and materials used to support the findings of this study are available from the corresponding authors (Benedetta Fibbi) upon request.

## Supplementary Materials

The following supporting information can be downloaded at: https://www.mdpi.com/article/10.3390/ijms242316680/s1.
