# Supplementary material for: Hyponatremia Promotes Cancer Growth in a Murine Xenograft Model of Neuroblastoma
Source: Int J Mol Sci. 2023 Nov 23;24(23):16680. doi: 10.3390/ijms242316680 (PMC10706371; doi:10.3390/ijms242316680)
Supplement: Supplementary file 1 [file ijms-24-16680-s001.zip › ijms-2684683-supplementary.pdf]

Supplementary Materials

Table S1. Tumor volume (mm^3) in control and hyponatremic mice, as measured at different time points. Results are espressed as mean ± SE.

	T0	T3	T6	T10	T12	T14	T20	T22	T26	T28
**control group (mm^3)**	60.4±123.7	106.5±22.38	233.7±64.8	576.5±242.5	807.5±308.4	1197.03±525.3	1543.2±402.5	1806.9±577.8	1790.6±303.2	2509.4±405.9
**hyponatremic group (mm^3)**	44.8±7.15	119.7±32.6	286.6±77.2	627.8±257.6	979.3±336.4	2136.8±988.03	2678.1±631.06	3164.7±839.3	2795.03±623.2	3692.5±817.7

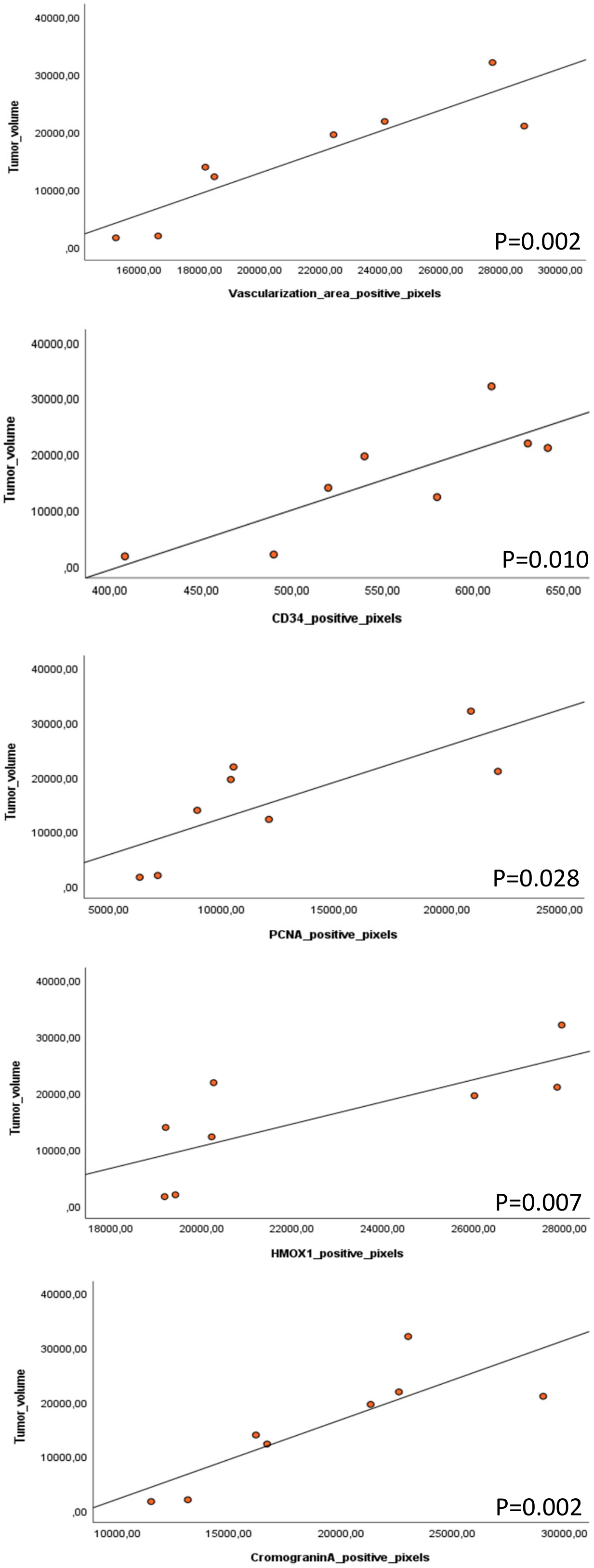

Tumor_volume
40000,00
30000,00
20000,00
10000,00
,00
P=0.002
16000,00 18000,00 20000,00 22000,00 24000,00 26000,00 28000,00 30000,00
Vascularization_area_positive_pixels
Tumor_volume
40000,00
30000,00
20000,00
10000,00
,00
P=0.010
400,00 450,00 500,00 550,00 600,00 650,00
CD34_positive_pixels
Tumor_volume
40000,00
30000,00
20000,00
10000,00
,00
P=0.028
5000,00 10000,00 15000,00 20000,00 25000,00
PCNA_positive_pixels
Tumor_volume
40000,00
30000,00
20000,00
10000,00
,00
P=0.007
18000,00 20000,00 22000,00 24000,00 26000,00 28000,00
HMOX1_positive_pixels
Tumor_volume
40000,00
30000,00
20000,00
10000,00
,00
P=0.002
10000,00 15000,00 20000,00 25000,00 30000,00
CromograninA_positive_pixels

(a)

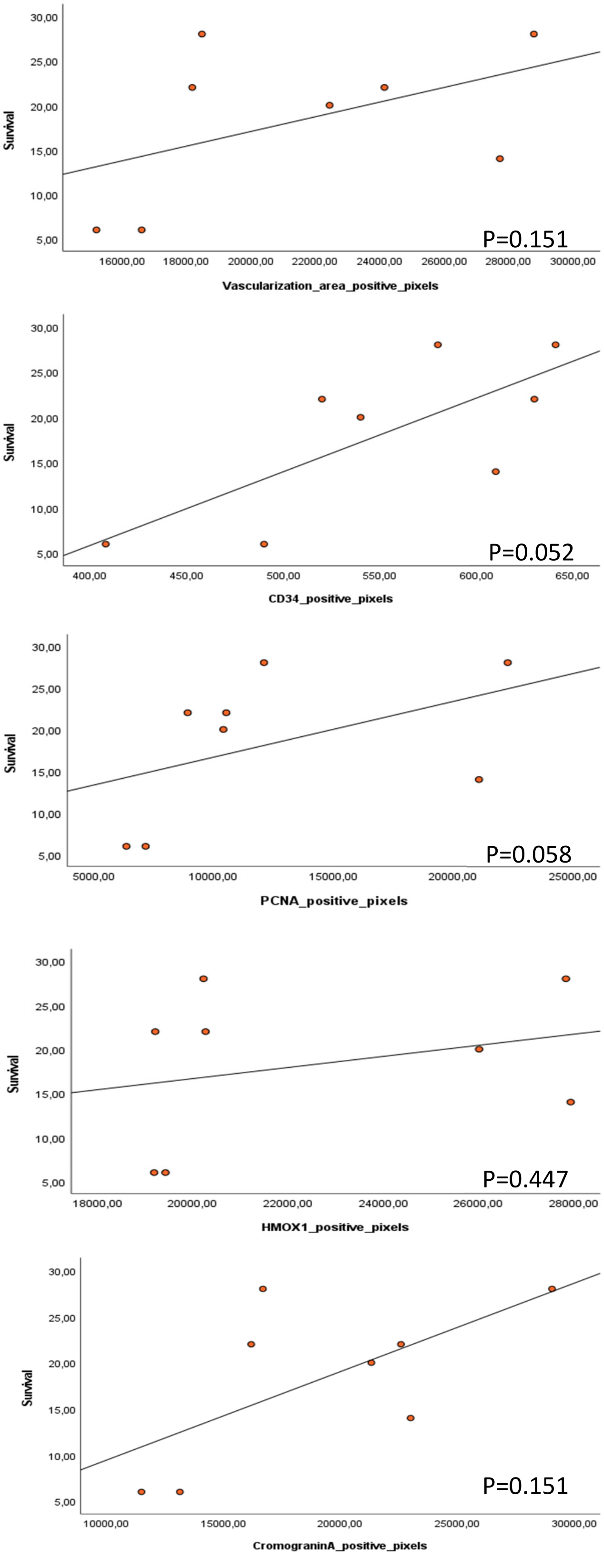

(b)

Figure S1 (a). Correlation analysis between tumor volume and histological markers; **(b)** correlation analysis between survival and histological markers.
